# Cytosolic Ca^2+^ transients during pulsed focused ultrasound generate reactive oxygen species and cause DNA damage in tumor cells

**DOI:** 10.7150/thno.48353

**Published:** 2021-01-01

**Authors:** Robert B. Rosenblatt, Joseph A. Frank, Scott R. Burks

**Affiliations:** 1 Frank Laboratory, Department of Radiology and Imaging Sciences, NIH Clinical Center, Bethesda, MD, 20892; 2 National Institute of Biomedical Imaging and Bioengineering, Bethesda, MD 20892

**Keywords:** focused ultrasound, calcium, DNA damage, tumor, reactive oxygen species

## Abstract

Mechanical forces from non-ablative pulsed focused ultrasound (pFUS) generate pro-inflammatory tumor microenvironments (TME), marked by increased cytokines, chemokines, and trophic factors, as well as immune cell infiltration and reduced tumor growth. pFUS also causes DNA damage within tumors, which is a potent activator of immunity and could contribute to changes in the TME. This study investigated mechanisms behind the mechanotransductive effects of pFUS causing DNA damage in several tumor cell types.

**Methods:** 4T1 (murine breast tumor), B16 (murine melanoma), C6 (rat glioma), or MDA-MB-231 (human breast tumor) cells were sonicated *in vitro* (1.1MHz; 6MPa PNP; 10ms pulses; 10% duty cycle; 300 pulses). DNA damage was detected by TUNEL, apoptosis was measured by immunocytochemistry for cleaved caspase-3. Calcium, superoxide, and H_2_O_2_ were detected by fluorescent indicators and modulated by BAPTA-AM, mtTEMPOL, or Trolox, respectively.

**Results:** pFUS increased TUNEL reactivity (range = 1.6-2.7-fold) in all cell types except C6 and did not induce apoptosis in any cell line. All lines displayed cytosolic Ca^2+^ transients during sonication. pFUS increased superoxide (range = 1.6-2.0-fold) and H_2_O_2_ (range = 2.3-2.8-fold) in all cell types except C6. BAPTA-AM blocked increased TUNEL reactivity, superoxide and H_2_O_2_ formation, while Trolox also blocked increased TUNEL reactivity increased after pFUS. mtTEMPOL allowed H_2_O_2_ formation and did not block increased TUNEL reactivity after pFUS. Unsonicated C6 cells had higher baseline concentrations of cytosolic Ca^2+^, superoxide, and H_2_O_2_, which were not associated with greater baseline TUNEL reactivity than the other cell lines.

**Conclusions:** Mechanotransduction of pFUS directly induces DNA damage in tumor cells by cytosolic Ca^2+^ transients causing formation of superoxide and subsequently, H_2_O_2_. These results further suggest potential clinical utility for pFUS. However, the lack of pFUS-induced DNA damage in C6 cells demonstrates a range of potential tumor responses that may arise from physiological differences such as Ca^2+^ or redox homeostasis.

## Introduction

Non-invasive image-guided focused ultrasound (FUS) is a therapeutic modality that has potential to precisely target and treat solid tumors with minimal bystander effects on intervening tissues 1. FUS has received FDA approval for thermal ablation of uterine fibroids and prostate tumors, and is currently in clinical trials as a potential adjuvant in tumor treatment, including immunotherapy 2, 3. Nonthermal pulsed FUS (pFUS) at high intensities mechanically ablates tissue (i.e., histotripsy) and has shown promise in preclinical studies to both ablate tumor volumes and induce antigen release that could stimulate innate and adaptive immune responses 4. Nondestructive sonication of tissues with pFUS at lower intensities can induce a wide range of molecular responses that occur through mechanotransduction of pFUS acoustic radiation forces (ARF) and cavitation 5.

pFUS, with or without microbubbles (MB), has been shown to activate inflammatory signaling pathways such as nuclear factor kappa-light-chain-enhancer of activated B cells (NFκB), tumor necrosis factor-α (TNFα), and cyclo-oxygenase 2 (COX2) signaling pathways 6-9 that can result in release of cytokines, chemokines, and trophic factors (CCTF) from cells in tissues into the surrounding microenvironments of normal and diseased tissues 9-20. Non-ablative pFUS alters CCTF and vascular cell adhesion molecule (CAM) expression for 24-48 h post-sonication in muscle 9-12, kidney 7, 13-15, heart 16, pancreas 17, brain 8, 18, and various malignancies 19, 20. Microenvironmental changes from nondestructive pFUS prior to infusion of cellular products increases tropism to targeted sites to prevent or improve tissue damage 7, 9, 11-15, 21-24. We recently reported that pFUS at a peak negative pressure (PNP) of 6 MPa (*f*_0_ = 1.15 MHz; no MB contrast agents) to murine flank tumor models of B16 melanoma and 4T1 breast tumors resulted in numerous proteomic changes to the tumor microenvironment (TME) that were accompanied by infiltration of immune cells into tumors 19, 20. Moreover, non-ablative pFUS generated a shift toward an anti-tumor TME and reduced growth rates of B16 and 4T1 flank tumors over time 20. Histological evaluation of possible mechanisms behind the retarded growth rates of the flank tumors following pFUS revealed increased nuclear DNA damage using terminal deoxynucleotidyl transferase dUTP end-nick labeling (TUNEL) reactions 19, 20. This observation was of particular interest as DNA damage and repair process can stimulate the innate immune system and alter the TME 25. Further, cellular release of DNA after apoptosis is a damage associated molecular pattern (DAMP) that can contribute to a pro-inflammatory TME and anti-tumor immune responses 26, 27.

The importance of DNA damage as a potential immune modulator following pFUS led us to investigate the mechanisms underlying sonication-induced DNA damage. Mechanical pFUS forces (ARF or cavitation) generates cytosolic Ca^2+^ transients in several cell types 6, 28-33 by activating mechanically-sensitive ion channels, such as the transient receptor potential (TRP) channels 6, 32, 34. Changes in cytosolic Ca^2+^ alter mitochondrial biology and generate reactive oxygen species (ROS) that can ultimately induce DNA damage 35. Our previous tumor studies 19, 20 did not mechanistically investigate how pFUS caused DNA damage or how it might contribute to slowing tumor growth. This study investigated the biological mechanisms underpinning pFUS-induced DNA damage *in vitro* in multiple tumor cell lines: B16-F10 (B16), a murine melanoma line; 4T1, a murine breast tumor line; C6, a rat glioma line; and MDA-MB-231BRL a luciferase-transfected human breast tumor line with high brain metastatic potential. Measurements of cytosolic Ca^2+^, ROS production, DNA damage, and apoptosis following pFUS were performed with and without various physiological manipulations to elucidate biological mechanisms. The results of this study also revealed different responses to pFUS across cell lines that were associated with underlying differences in cell biology and physiology that could ultimately influence how non-ablative pFUS should be incorporated into the treatment of malignancy.

## Materials and Methods

### Cell Culture

4T1, MDA-MB-231, B16, and C6 were all cultured in Roswell Park Memorial Institute (RPMI) 1640 medium without phenol red and supplemented with 10% FBS and 1% penicillin/streptomycin. Cells were grown at 37 ºC under 5% CO_2_ atmosphere. 4T1, B16, and C6 cells were from ATCC (Manassas, VA). MDA-MB-231 were the brain-metastasizing subtype previously transfected with a luciferase reporter gene and were a gift from Dr. Patricia Steeg (National Cancer Institute, Bethesda, MD).

### Pulsed Focused Ultrasound

pFUS was delivered with a focused transducer (H101, Sonic Concepts, Bothell WA) operating at 1.1 MHz and driven with hardware controlled by LabView (National Instruments, Austin TX). All suspended-cell samples were treated using 6 MPa peak negative pressure (PNP), 10 ms pulse length, 10 Hz pulse repetition frequency, and 300 pulses. These parameters were previously used *in vivo* 19, 20 and PNP were measured in degassed water. Treatments were performed with cells suspended in RPMI-1640 in 500 μL centrifuge tubes. Cell suspensions were placed at the focus of the transducer submerged in degassed water. Untreated cell groups were received sham sonications (power=0 W). For live-cell Ca^2+^ imaging, the transducer was placed in a coupling cone filled with degassed water and aligned over the objective of an inverted epifluorescence microscope (Axio Observer 5, Zeiss, Oberkochen, Germany). Cells were adhered to 35mm dishes with #1 glass bottoms and the transducer cone was coupled directly into the imaging medium during sonication. Sonication for live-cell Fluo-4 imaging (Figure 2A-B) was performed at 3 MPa to minimize detachment or movement of cells during image acquisition.

### Intracellular Loading with Chemicals to Modulate Physiology and Fluorescent Indicators

For some experiments, cells were incubated at room temperature with various agents including: 1,2-bis(o-aminophenoxy) ethane-N,N,N',N'-tetraacetic acid-acetoxymethyl ester (BAPTA-AM) to chelate intracellular calcium at 1μM for 30 min; 2-(2,2,6,6-Tetramethylpiperidin-1-oxyl-4-ylamino)-2-oxoethyl) triphenylphosphonium chloride (mtTEMPOL) to neutralize superoxide at a 20 μM for 1 h; or (±)-6-Hydroxy-2,5,7,8-tetramethylchromane-2-carboxylic acid (Trolox), a peroxyl radical scavenger at 5 μM for 30 minutes. All incubations were performed prior to pFUS.

Fluorescent indicators were also loaded into cells. Cells cultured in glass-bottomed imaging dishes were loaded with Fluo-4 AM to detect intracellular calcium (ThermoFisher, Waltham, MA) according to manufacturer instructions prior to pFUS. Suspended cells were incubated with either MitoSOX (mitochondrial superoxide indicator) (ThermoFisher) at 5 μM immediately prior to pFUS or a proprietary intracellular H_2_O_2_ indicator (Cat# MAK164; MilliporeSigma, St. Louis, MO) immediately after pFUS according to manufacturer instructions. Cells loaded with MitoSOX or the H_2_O_2_ indicator were re-plated into glass-bottomed dishes 2 h prior to imaging.

### TUNEL and Immunocytochemistry (ICC)

Cells for TUNEL measurements were re-plated following treatment and incubated for 6 hr. Cells were fixed in 4% paraformaldehyde for 1 h at 4 ºC and permeabilized with 0.1% Triton X-100 for 10 minutes. For TUNEL quantification, ~10^5^ cells in 96-well plates were measured using fluorescence-based In Situ Cell Death Detection Kit (Millipore Sigma) according to manufacturer guidelines. Cells were incubated with 4′,6-diamidino-2-phenylindole (DAPI) and fluorescence of TUNEL substrates and DAPI were measured using a plate reader. TUNEL reactivity was quantified as the ratio of fluorescent TUNEL intensity to DAPI intensity. For TUNEL imaging, cells were spun onto glass slides using the Thermo Shandon Cytospin 3 and then fixed and permeabilized. TUNEL reactions were performed on slides and imaged with an upright fluorescent microscope (Axio, Zeiss).

Immunocytochemistry (ICC) for activated caspase-3 was performed on fixed and permeabilized cells. Slides were blocked with SuperBlock (ThermoFisher) for 30 minutes and then cells were incubated for 1 h with an anti-Caspase 3 (cleaved form) antibody (Cat# PC679; Millipore Sigma) at a dilution of 1:100. The secondary antibody was an AlexaFluor 555-conjugated donkey anti-rabbit antibody (Invitrogen) incubated at a dilution of 1:1000 for 30 minutes. Cells were then imaged using an upright fluorescent microscope. Positive control groups for both TUNEL and activated caspase-3 measurements consisted of re-plated cells being incubated for 6 h in the presence of 1 mM H_2_O_2_. This reflects the duration that H_2_O_2_ formation would occur for following sonication of pFUS-treated groups.

### Cytosolic and Intracellular Calcium Measurements

Suspensions containing 2 × 10^5^ cells/mL were permeabilized in divalent-ion-free phosphate buffered saline (PBS) containing 20 μM digitonin or 0.1% (v/v) Triton X-100. Cellular debris were centrifuged and Fluo-4, Pentapotassium Salt (1 μM), was added to the supernatant. Fluorescence was measured on a fluorometric plate reader and compared to a standard curve of CaCl_2_. Cell diameters were measured for each cell line by microscopy (Figure S1) and spherical intracellular volumes were calculated. Ca^2+^ concentrations from supernatants were adjusted based on intracellular volumes to approximate cytosolic Ca^2+^ concentrations.

### Image and Data Analyses

Image analyses for activated caspase-3, Fluo-4, and the H_2_O_2_ and superoxide (MitoSOX) indicators were processed using ImageJ (v2.0). Using controls (i.e., no added dye), thresholding was applied to remove background and the mean cellular fluorescence was measured in images. Regions-of-interest (ROI) were drawn around individual cells for Fluo-4 imaging while mean cellular fluorescence of activated caspase-3, the H_2_O_2_ and superoxide indicators were measured in the entire microscopic frames. Statistical analyses and data presentation were performed in Prism (v8, GraphPad, La Jolla, CA). All figures present means and standard deviations. Statistical testing used t-tests for pairwise comparisons and one-way analyses of variance (ANOVA) for multiple comparisons. All statistical tests were two-sided and p values <0.05 were considered significant.

## Results

### pFUS increases TUNEL reactivity in cancer cells without inducing apoptosis

**Figure 1:**
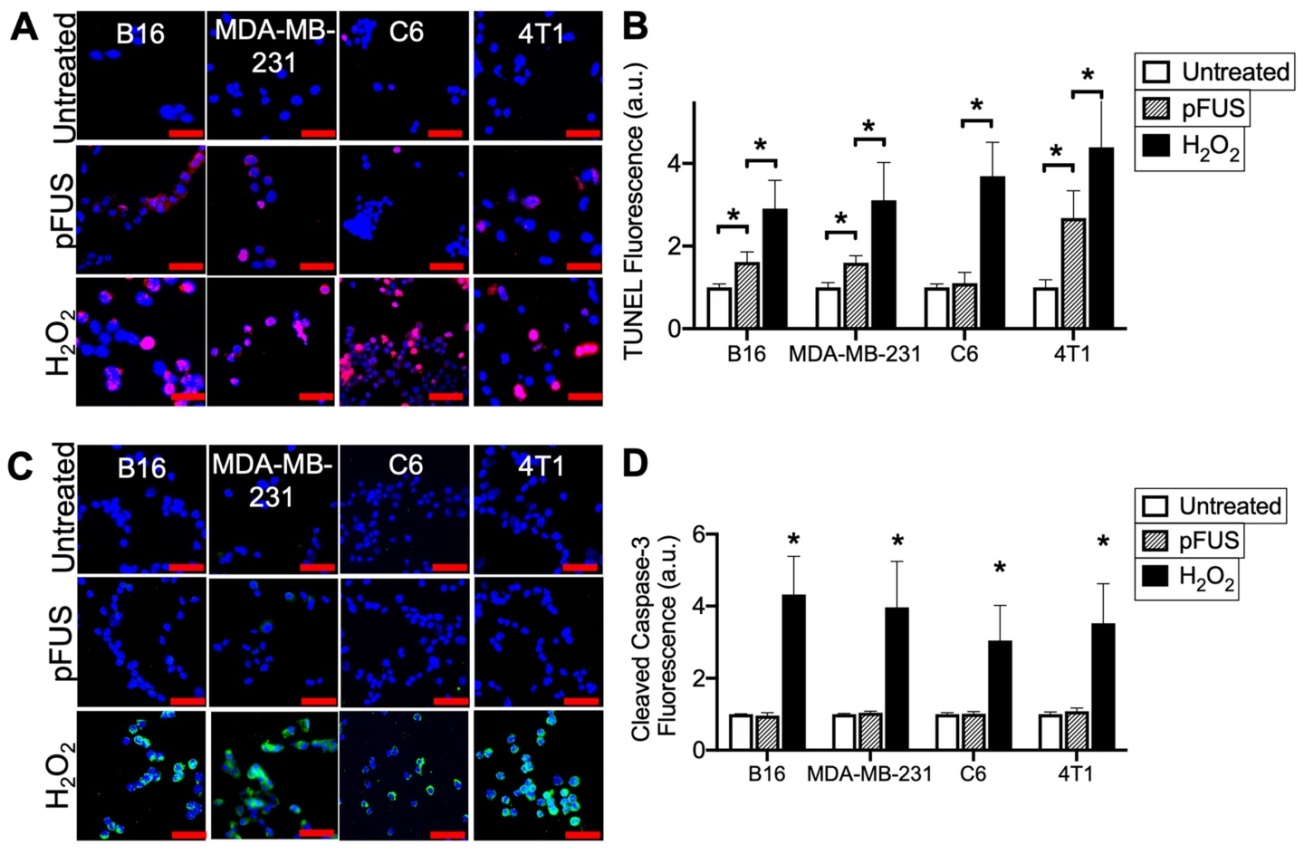
** pFUS increases TUNEL reactivity without apoptosis in tumor cells.** A) Representative imaging (TUNEL-positive nuclei in red) and B) quantification of TUNEL reactivity in tumor cells with or without pFUS (n = 9 per group per cell line) at 6 h post-pFUS. C) Representative imaging of ICC for cleaved (activated) caspase-3 (green) in tumor cells with or without pFUS at 6 h post-pFUS. D) Quantification of activated caspase-3 in each group (n = 9 per group per cell line). H_2_O_2_ group in each panel represents a positive controls for each measurement where cells were incubated with H_2_O_2_ (1 mM) for 6 h. Asterisks represent p < 0.05 by ANOVA comparisons performed on all groups for each cell line. Scale bars = 50 μm.

Cells were sonicated with pFUS operating at 1.1 MHz using 6 MPa PNP, 10 ms pulse length, 10 Hz pulse repetition frequency, and 300 pulses. Sonication increased nuclear TUNEL reactivity at 6 h in the B16, MDA-MB-231 and 4T1 cells (p < 0.05 by ANOVA) (N = 9 per per cell line) (Figure 1A-B). There was no increase in nuclear TUNEL reactivity detected following pFUS to C6 cells. A positive control incubating cells with H_2_O_2_ (1 mM) for 6 h prior to fixation revealed increased DNA damage in all cell lines (p < 0.05 by ANOVA). Although increased TUNEL reactivity was observed in some cell lines, ICC revealed that pFUS did not increase activation of caspase-3, an indicator of apoptosis, however apoptosis was significantly induced in all lines following incubation with H_2_O_2_ (p < 0.05 by ANOVA) (Figure 1C).

### pFUS generates cytosolic Ca^2+^ transients and increased TUNEL reactivity is suppressed by intracellular Ca^2+^ chelation

**Figure 2:**
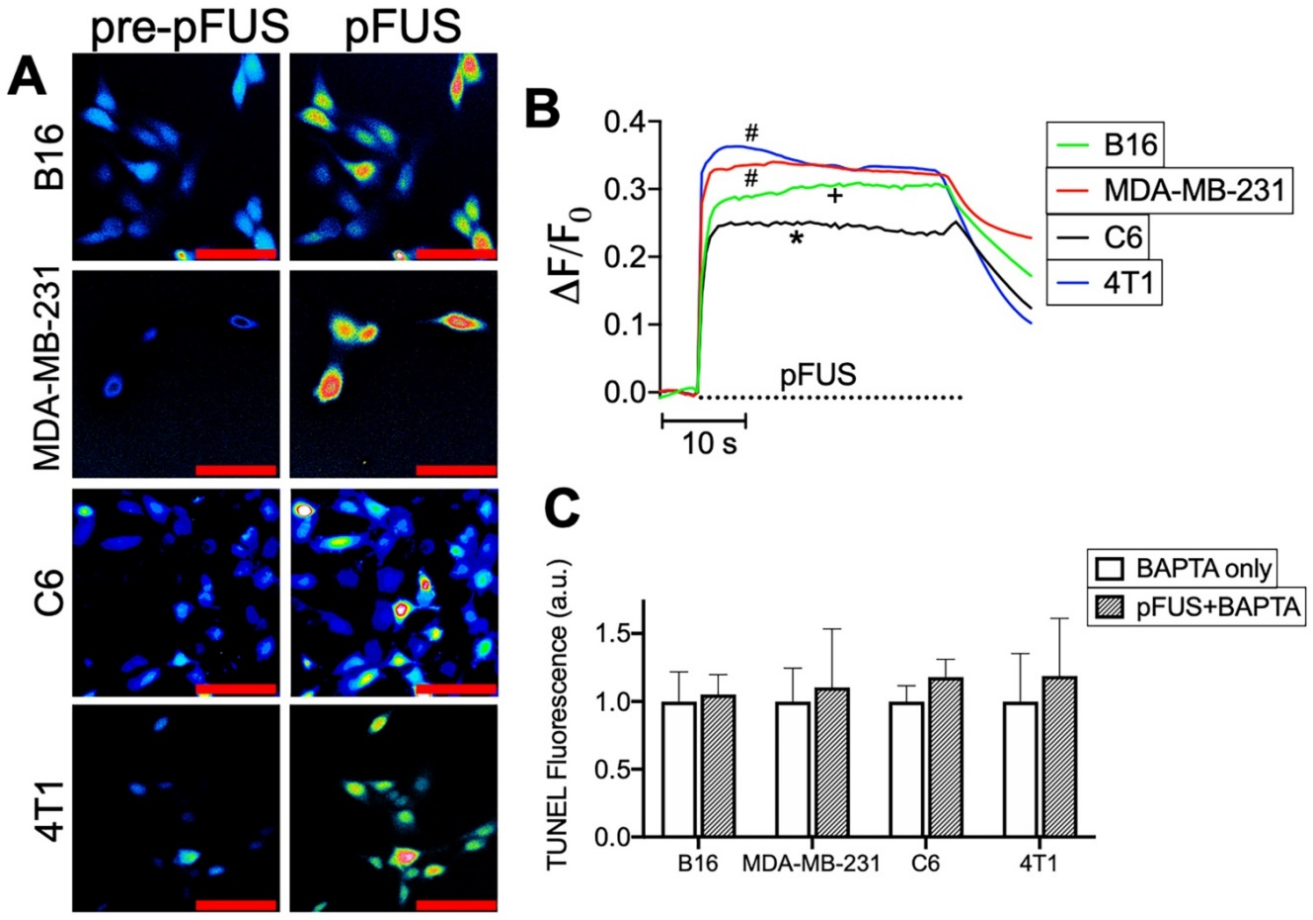
** pFUS activates cytosolic Ca^2+^ transients in tumor cells and intracellular Ca^2+^ chelation suppresses pFUS-induced TUNEL reactivity.** A) Representative imaging of Fluo-4 fluorescence before and during sonication (psuedocolor; scale bars = 50 μm) and B) fluorometric traces of Fluo-4 intensity before and during sonication (n = 69-130 per cell type; dashed line represents sonication time). Groups with like symbols are statistically similar to each other and significantly different from groups denoted by other symbols following ANOVA comparing peak magnitudes of each group. C) Quantification of TUNEL reactivity in cells with or without pFUS in the presence of intracellular BAPTA (n = 9 per group per cell type). Statistical significance was tested between control and treated groups using t-tests for each cell line.

To delineate the potential relationship between pFUS and TUNEL reactivity, cytosolic Ca^2+^ entry during sonication was determined by incubating cells with the Ca^2+^ ionophore Fluo-4-AM. Live cell imaging revealed cytosolic Ca^2+^ influxes in all cell lines (n = 64-130 cells per cell line) that persisted through the duration of pFUS treatment (Figure 2A-B). Multiple comparisons of maximal ΔF/F_0_ values by ANOVA revealed that MDA-MB-231 and 4T1 lines exhibited the greatest mean changes in fluorescence intensities and were statistically similar to each other (p > 0.05). The B16 line was significantly lower (p < 0.05) than either MDA-MB-231 or 4T1 cells. C6 cells exhibited significantly smaller changes (p < 0.05) than all other cell lines. In order to define the influence of cytosolic Ca^2+^ transients on TUNEL reactivity, cells were incubated with the Ca^2+^ chelator BAPTA-AM prior to pFUS treatments. BAPTA-loaded cells were measured for TUNEL reactivity at 6 h (n = 9 per cell line). BAPTA prevented pFUS from increasing TUNEL reactivity and were statistically similar to BAPTA -loaded sham-sonicated cells (p > 0.05 by t-tests) (Figure 2C).

### pFUS induces Ca^2+^-dependent mitochondrial superoxide formation, but increased TUNEL reactivity is not suppressed by a superoxide dismutase (SOD) mimetic

**Figure 3:**
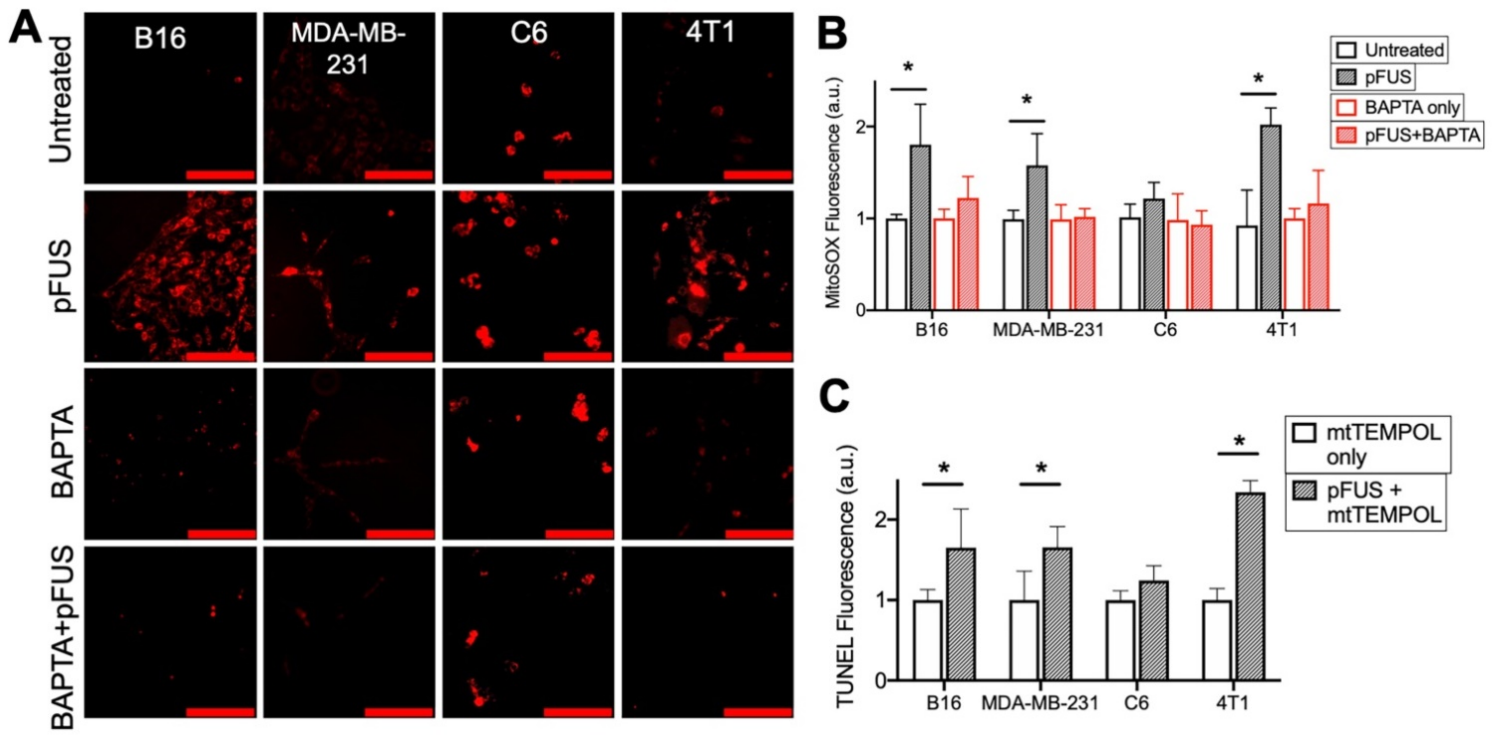
** pFUS increases Ca^2+^-dependent superoxide formation in cells, but neutralization of superoxide by SOD mimetic does not reduce pFUS-induced increases in TUNEL reactivity.** A) Representative imaging of Mitosox fluorescence intensity (red; scale bars = 100 μm) and B) quantification with or without pFUS and in the presence or absence of intracellular BAPTA at 2 h post-sonication (n = 15 per group per cell line). Mitosox was loaded immediately prior to pFUS. Asterisks represent p < 0.05 from ANOVA comparisons performed on all groups from each cell line. C) Quantification of TUNEL reactivity with or without pFUS following incubation with the SOD mimetic mtTEMPOL (n = 9 per group per cell line). Asterisks represent p < 0.05 by t-tests between treated and control groups for each cell line.

To investigate the role of cytosolic Ca^2+^ influx and mitochondrial superoxide formation generated by pFUS, cells were incubated with MitoSOX immediately before sonication. Fluorescence quantification from live-cell imaging (n = 15 per cell line) at 2 h post-pFUS revealed significantly increased (p < 0.05 by ANOVA) superoxide production in the B16, MDA-MB-231, and 4T1 cell lines, but not in the C6 line (p > 0.05 by ANOVA) (Figure 3A-B). Chelation of cytosolic Ca^2+^ by BAPTA did not affect baseline levels of superoxide production (p > 0.05 by ANOVA) but, did prevent pFUS from significantly increasing superoxide production in any cell line compared to controls (p > 0.05 by ANOVA).

The effect of mitochondrial superoxide on TUNEL reactivity post- pFUS was investigated. Cells were incubated with the mitochondrial-targeting SOD mimetic mtTEMPOL (20 μM) prior to pFUS (n = 9 per cell line). mtTEMPOL effectively neutralized superoxide formation following pFUS treatment (Figure S1). However, mtTEMPOL did not significantly (p < 0.05 by t-test) block pFUS-induced increases in TUNEL reactivity for the B16, MDA-MB-231, and 4T1 cell lines after 6 h compared to control cells (Figure 3C). TUNEL reactivity for C6 cells did not increase following pFUS in the presence of mtTEMPOL (p > 0.05 by t-test).

### pFUS induces Ca^2+^-dependent H_2_O_2_ formation and increased TUNEL reactivity is suppressed by peroxyl radical scavengers

**Figure 4:**
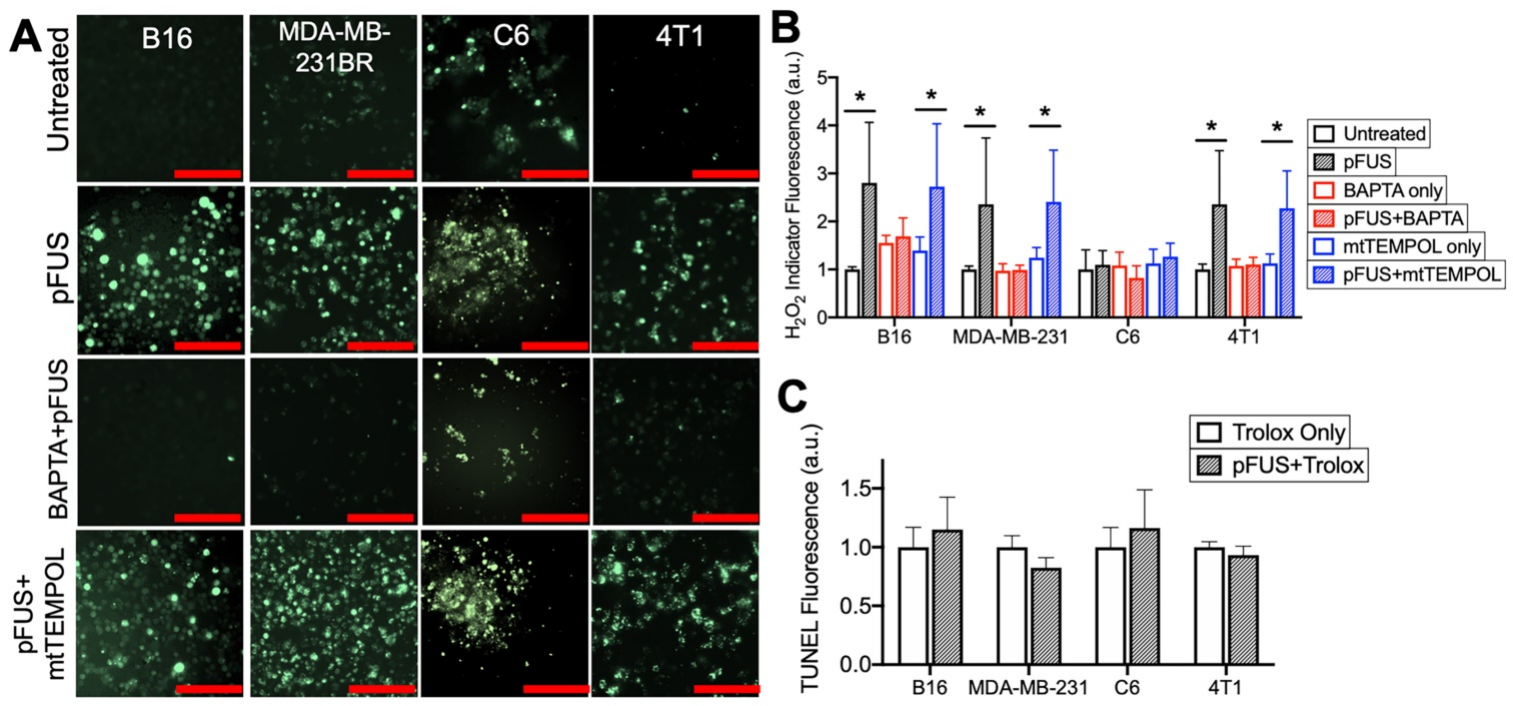
** pFUS increases Ca^2+^-dependent formation of H_2_O_2_ and neutralization of H_2_O_2_ with Trolox suppresses pFUS-induced increases in TUNEL reactivity.** A) Representative images of intracellular H_2_O_2_ indicator (MAK-164, MilliporeSigma) fluorescence (green; scale bars = 100 μm) and B) quantification with or without pFUS and in the presence or absence of intracellular BAPTA or mtTEMPOL at 2 h post-sonication. The H_2_O_2_ indicator was loaded immediately after pFUS. Asterisks represent p < 0.05 by ANOVA comparisons performed on all groups from each cell line. C) Quantification of TUNEL reactivity in cells with or with pFUS in the presence of intracellular Trolox (n = 9 per group per cell type). Statistical significance was tested between control and treated groups using t-tests for each cell line.

SOD catalyzes the formation H_2_O_2_ radicals and therefore, H_2_O_2_ formation and its potential role in pFUS-induced TUNEL reactivity was investigated. Intracellular H_2_O_2_ was detected using a fluorescent indicator loaded into cells immediately after pFUS (n = 15 per cell type). The B16, MDA-MB-231, and 4T1 cells showed significantly increased H_2_O_2_ formation at 2 h post-pFUS compared to controls (p < 0.05 by ANOVA) (Figure 4A-B). The C6 line showed no significant increase in H_2_O_2_ formation following pFUS (p > 0.05 by ANOVA).

Chelating cytosolic Ca^2+^ with BAPTA (n = 15 per cell type) did not affect baseline H_2_O_2_ formation in any cell line (p > 0.05 by ANOVA), but it did suppress increased H_2_O_2_ formation following pFUS treatment in the B16, MDA-MB-231 and 4T1 lines (p < 0.05 by ANOVA) (Figure 4A-B). No significant increase in H_2_O_2_ formation was observed in sonicated C6 cells loaded with BAPTA (p < 0.05 by ANOVA). pFUS-induced H_2_O_2_ formation was also investigated in the presence of mtTEMPOL (20 μM) (n = 15 per cell type). The B16, MDA-MB-231 and 4T1 lines incubated with mtTEMPOL still demonstrated significant increases in H_2_O_2_ formation following pFUS (p < 0.05 by ANOVA) while the C6 line showed no increase in H_2_O_2_ formation following pFUS (p > 0.05).

Lastly, each cell line was incubated with the peroxyl radical scavenger Trolox (5 μM) prior to pFUS treatment and assayed for TUNEL reactivity at 6 h post-sonication (n = 9 per cell line). The presence of Trolox inhibited increased TUNEL reactivity in the B16, MDA-MB-231 and 4T1 lines following pFUS. Additionally, the C6 line exhibited no statistical increase in TUNEL reactivity in the presence of Trolox (p > 0.05 by ANOVA).

### Physiological differences between tumor cell lines

**Figure 5:**
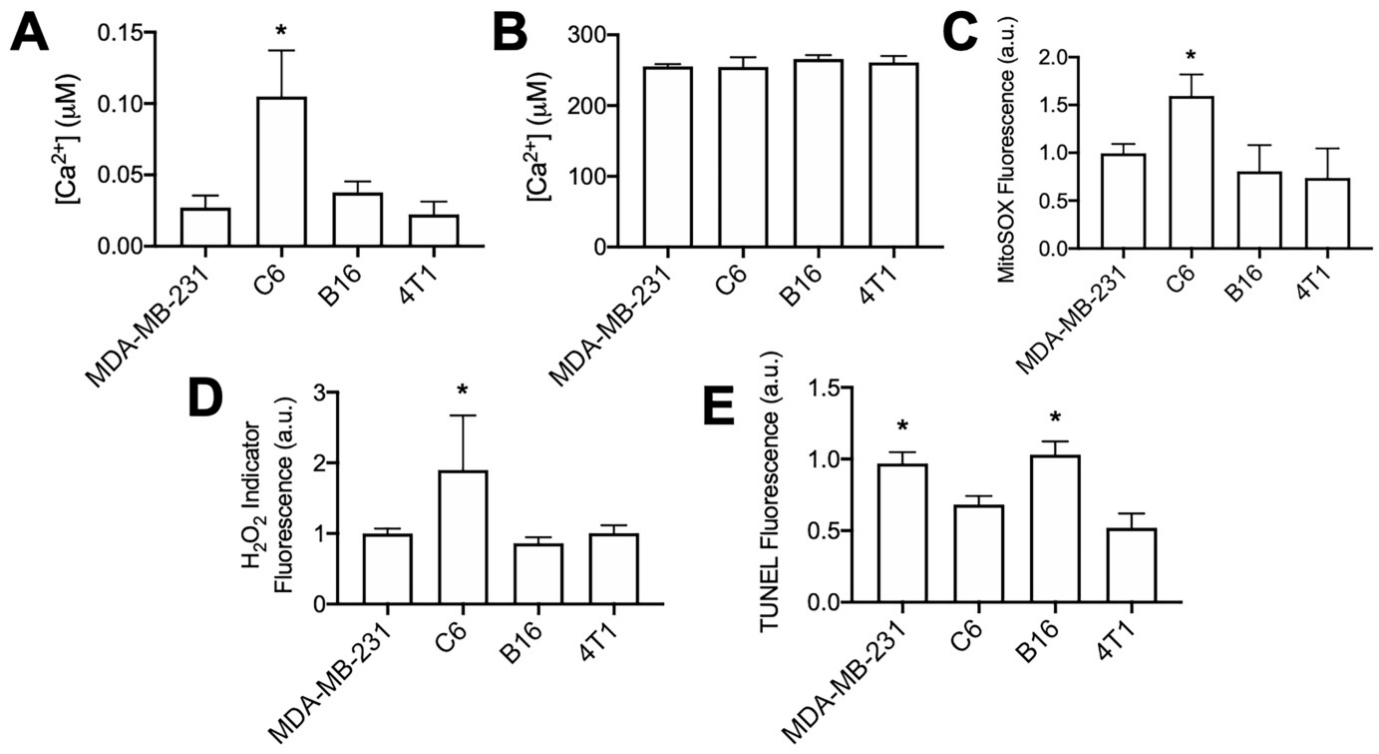
** Unsonicated C6 cells have elevated concentrations of cytosolic Ca^2+^, superoxide, and H_2_O_2_ compared to other unsonicated cells types, not higher levels of TUNEL reactivity.** A) Ca^2+^ quantification in cytosolic volumes by Fluo-4 following cell permeabilization with digitonin (20 μM) (n = 8-9 per cell type). B) Ca^2+^ quantification in intracellular volumes by Fluo-4 following cell permeabilization with Triton X-100 (0.1% v/v) (n = 5 per cell type). C) Quantification of Mitosox fluorescence following 2 h intracellular incubation (n = 15 per cell type). D) Quantification of intracellular H_2_O_2_ indicator (MAK-164, MilliporeSigma) fluorescence following 2 h intracellular incubation (n = 15 per cell type). E) Quantification of TUNEL reactivity in all cell types without sonication (n = 9 per cell type). Asterisks in all graphs represent p < 0.05 by ANOVA for each measurement. Groups with asterisks in (E) were statistically similar (p < 0.05).

The C6 cell line demonstrated markedly different responses to pFUS than other tumor cell lines assayed in this study. Although cytosolic Ca^2+^ transients were observed during pFUS, it was unable to increase superoxide, H_2_O_2_, or TUNEL reactivity in C6 cells. C6 cells demonstrated the smallest relative increase in Fluo-4 fluorescence during pFUS, but those relative measurements provide little information regarding the resting cytosolic Ca^2+^ concentrations. Therefore, the cytosolic fractions from each cell line in the absence of pFUS were collected following permeabilization with digitonin (20 μM) in a divalent-ion-free solution. Lysates were incubated with Fluo-4 (pentapotassium salt; 1 μM) (n = 8-9 per cell line) and fluorescence intensities were compared to standard curves obtained with CaCl_2_ solutions. Cytosolic Ca^2+^ concentrations were estimated by adjusting values to average intracellular volumes measured for each cell line (Figure S2). C6 cells were revealed to have statistically higher resting cytosolic concentration of Ca^2+^ compared to the other cell lines (Figure 5A; p < 0.05 by ANOVA). All cell lines had similar intracellular Ca^2+^ concentrations when measuring lysates following permeabilization with Triton X-100 (0.1% v/v) to obtain organellular fractions (n = 5 per cell line).

Furthermore, experiments directly comparing each cell line at baseline (no pFUS) were performed to determine relative superoxide and H_2_O_2_ production among the cell lines (n = 15 per cell line). C6 cells demonstrated statistically elevated levels (p < 0.05 by ANOVA) of both superoxide (Figure 5B) and H_2_O_2_ (Figure 5C) compared to the three other cell lines. Lastly, samples of each cell line (n = 8 per cell line) were directly compared for TUNEL reactivity without pFUS treatment. Increased cytosolic Ca^2+^, superoxide, and H_2_O_2_ levels in C6 cells did not correlate with higher baseline TUNEL reactivity (Figure 5D) compared to other cell lines. The B16 and MDA-MB-231 lines exhibited the highest baseline levels of TUNEL reactivity and were statistically similar to each other (p > 0.05 by ANOVA). Moreover, TUNEL reactivities in the C6 and 4T1 lines were statistically similar to each other (p > 0.05 by ANOVA) and lower than B16 and MDA-MB-231 (p < 0.05 by ANOVA).

## Discussion

This study demonstrates that pFUS can induce DNA damage in some tumor cell lines such as murine 4T1 breast tumor, B16 melanoma and human MDA-MB-231 breast tumors, but not rat C6 glioma. DNA damage occurred after pFUS without initiating apoptosis. DNA damage resulted from pFUS eliciting cytosolic Ca^2+^ transients that increased mitochondrial superoxide formation and subsequent H_2_O_2_ formation. The lack of pFUS effects and inherent physiological differences of the C6 demonstrated that differences in susceptibility to pFUS exist among different tumor cell lines.

In this study, the *in vitro* culture systems allowed physiological measurements that are difficult in complex *in vivo* tumor models. Furthermore, sonicating tumor cells alone can determine whether the DNA damage observed in previous *in vivo* studies was a direct result of pFUS or a bystander effect from the anti-tumor shift in the TME and immune system activation. This study did not rule out potential contributions from the CCTF and immune cell populations in the TME contributing to DNA damage observed in non-ablative flank tumor studies 19, 20. However, it demonstrated that pFUS alone at the described parameters was sufficient to cause DNA damage by TUNEL assays without apoptosis. Due to the complexity of DNA damage/repair mechanisms, apoptosis is not always the consequence of DNA damage 36. Due to these complexities and potential differences between cell lines, this study measured direct DNA damage by TUNEL as opposed to other methods (*e.g.* γ-H2AX staining) that are associated DNA repair process which could confound interpretations.

pFUS generated cytosolic Ca^2+^ transients during sonication which were consistent with previous studies in normal tissues 6. Live-cell Fluo-4 imaging was performed at 3 MPa to permit high-quality image acquisition and sonicated cells cultured on a glass substrate rather than in suspension. Thus, there is some difficulty extrapolating relative differences in cytosolic Ca^2+^ transients at 3 MPa to cells treated at 6 MPa in all other experiments. However, the necessity of increased cytosolic Ca^2+^ during pFUS at 6 MPa was clearly demonstrated by chelating intracellular Ca^2+^ with BAPTA. Increased DNA damage was not apparent in C6 glioma despite generating cytosolic Ca^2+^ transients. pFUS-induced DNA damage depended on cytosolic Ca^2+^ entry, but it remains unclear specifically how pFUS affects intracellular Ca^2+^ dynamics. Cytosolic Ca^2+^ increases during sonoporation by ultrasound with MB contrast agents have been widely reported 37-39. Sonoporation allows diffusion of Ca^2+^ into the cytosol through indiscriminate openings of the plasma membrane and can induce Ca^2+^-dependent bioeffects 38. However, we previously demonstrated 6 that pFUS at 4 MPa without MB opened mechano-sensitive transient receptor potential C1 (TRPC1) channels in the plasma membranes of normal kidney and skeletal muscle cells. Na^+^- and Ca^2+^-containing currents through TRPC1 channels activate complexed voltage-gated Ca^2+^ channels (VGCC) to amplify Ca^2+^ entry from the extracellular space. Future studies should examine specific mechanisms of cytosolic Ca^2+^ influx into tumor cells and investigate entry from both the plasma membrane and endoplasmic reticulum (ER) Ca^2+^ release through mechanisms such as Ca^2+^-induced Ca^2+^ release (CICR) or inositol triphosphate (IP_3_)-mediated Ca^2+^ release 40.

Increased cytosolic Ca^2+^ following pFUS initiates a cascade of biological processes. Cytosolic Ca^2+^ can generate mitochondrial Ca^2+^ flux through the mitochondrial Ca^2+^ uniporter (MCU) 41. Mitochondrial Ca^2+^ influx augments O_2_ metabolism and drives formation of ROS, including superoxide 35. Increased superoxide formation was observed in cell lines that exhibited pFUS-induced DNA damage and found to be dependent on elevated cytosolic Ca^2+^. To investigate the role of superoxide formation in pFUS-induced DNA damage, cells were loaded with a mitochondria-targeting antioxidant (mtTEMPOL) that acts as a SOD mimetic 42. mtTEMPOL effectively scavenged mitochondrial superoxide (see Figure S2). However, mtTEMPOL did not reduce the incidence of DNA damage in pFUS-treated cells, which would presumably indicate a lack of involvement of contributing to DNA damage. SOD and mtTEMPOL catalyze a single-electron reduction of superoxide leading to the formation of the ROS H_2_O_2_ 43. Superoxide, while thus a necessary intermediate, is membrane impermeant and would not escape the mitochondria to interact with nuclear DNA. Therefore, we investigated the role for pFUS to increase H_2_O_2_ formation via superoxide dismutation and its potential relationship to DNA damage.

Increased H_2_O_2_ formation following pFUS was detected in the three cell lines that exhibited DNA damage following pFUS. H_2_O_2_ formation was suppressed by chelating intracellular Ca^2+^, but not by the SOD mimetic. Since the SOD mimetic did not prevent pFUS-induced H_2_O_2_ formation it is possible that the changes in H_2_O_2_ could be involved in DNA damage. To test this hypothesis, cells were incubated with Trolox to neutralize H_2_O_2_ radicals 44 and assessed DNA damage following pFUS. Trolox suppressed increases in TUNEL reactivity following pFUS and demonstrated that H_2_O_2_ contributed to DNA damage in sonicated tumor cells. H_2_O_2_ is freely diffusible throughout the cell and H_2_O_2_ can cause nuclear DNA damage after undergoing Fenton reactions 45. The TUNEL assay detects double-strand DNA breaks and while controversial, H_2_O_2_ can result in double-strand DNA breaks 46. Additionally, DNA replication is frequently upregulated in tumor cells 47 and single-strand breaks from H_2_O_2_ on a complementary strand during replication would be detectable by TUNEL.

In the current study, C6 glioma cells appeared resistant to pFUS-induced increases in ROS production or DNA damage. pFUS increased cytosolic Ca^2+^ in C6 cells, but this did not translate into increased superoxide or H_2_O_2_ formation. Physiological profiling of the different cell types in their native states revealed that C6 cells had significantly higher levels of cytosolic Ca^2+^, superoxide, and H_2_O_2_. We hypothesize that elevated baseline levels of ROS would be due to elevated cytosolic Ca^2+^ concentrations causing increased mitochondrial Ca^2+^ through MCU transport. One explanation for the inability of pFUS to increase ROS through cytosolic Ca^2+^ transients is potentially abnormal MCU expression in C6 cells. This study did not examine mitochondrial expression of MCU in these cell lines, but human gliomas have lower MCU expression compared to other tumor types 48, 49. It is feasible that elevated baseline cytosolic Ca^2+^ concentrations combined with lower MCU expression causes mitochondrial Ca^2+^ transport to be at or near saturation in C6 cells. Thus, the elevation in cytosolic Ca^2+^ from pFUS does not substantially increase mitochondrial Ca^2+^ flux and thus does not stimulate additional ROS formation or DNA damage. Interestingly, BAPTA alone did not reduce baseline ROS concentrations in C6 cells. This could result from the 2 h incubation period not being long enough to markedly reduce baseline ROS production. Previous studies have shown C6 cells to be sensitive to sonodynamic therapies 50, 51, which rely on Ca^2+^ overload. However, we hypothesize that magnitude and/or duration of increased Ca^2+^ between SDT and pFUS would be vastly different.

An additional observation to consider in C6 cells is that higher baseline ROS concentrations did not correlate with higher baseline TUNEL reactivity. It is possible that C6 glioma cells have inherent protection mechanisms from ROS-induced DNA damage aside from neutralizing ROS such as enhanced DNA repair processes. Previous studies in alveolar epithelium have demonstrated resistance to DNA damage in spite of chronically elevated H_2_O_2_ concentrations 52. Further studies are warranted on the unique nature of C6 cells to further understand their resistance to pFUS-induced DNA damage. These physiological observations may help explain the resistance of gliomas to radiation therapy 53 which causes tumor cell damage via ROS production and serve as indicators to identify tumors that have altered sensitivity to pFUS-induced DNA damage.

While the lack of C6 response to pFUS is notable from a potential therapeutic standpoint, the remaining cell lines showed desired responses to pFUS, including two breast tumor lines from different species. It is unclear if the similarities in breast cancer (and also melanoma) responses are simply coincidental, but they could be related to metastatic potential of those tumor lines. Previous research has demonstrated a role for the mechanostretch receptor TRPC1 in the invasion and metastatic potential of numerous tumors 54. While TRPC1 expression was not specifically assayed in this study, C6 gliomas have been demonstrated not to upregulate TRPC1 55. The potential necessity for TRPC1 to mediate pFUS-induced Ca^2+^ signaling at similar intensities to those used here 6 open the possibility of a correlation between pFUS responses and expression of mechanostrech receptors like TRPC1. Further investigations are warranted to examine the expression of TRPC1 or similar stretch-sensitive cationic channels and pFUS-induced DNA damage in different types of cancers and cell lines from different species.

The primary goal of this study was to investigate biological mechanisms behind DNA damage from pFUS, however the results have wide implications for potential clinical translation as an anti-tumor therapy for three main reasons. First, DNA damage may play a critical role in activating the immune system. While extracellular DNA is a DAMP 26, 27, DNA damage and repair processes in non-apoptotic cells can stimulate the immune system 25. Interestingly, this investigation also revealed H_2_O_2_ as a mediator of DNA damage. However, H_2_O_2_ also induces neutrophil recruitment as the first phase of innate immune response and could directly contribute to a shift towards a pro-inflammatory TME 56. Second, the lack of apoptosis is an initial demonstration that non-ablative pFUS may be possible to incorporate as an immunological adjuvant approach to treat peripheral tissues containing radiologically undetected local micro-metastatic disease. Non-destructive pFUS could be used to alter the microenvironment of normal-appearing tissue to trigger innate and adaptive immune responses in areas containing microscopic tumors while preserving the architecture and function of nonmalignant tissues. Our previous work has extensively demonstrated pFUS at 4 MPa does not have deleterious effects on normal and diseased muscle 9-12 or kidney 7, 13, 15 tissue, yet augments a pro-inflammatory microenvironment to increase homing of stem cells. Future studies would need to be performed at different pFUS intensities to see if optimal parameters could be identified to mount anti-tumor responses in regions containing radiologically undetectable disease without negatively impacting surrounding normal tissues. Lastly, not all tumor types may be responsive to the mechanical effects of pFUS. The results of this study suggest that inherent biological differences between tumor cell types could render some tumors more or less sensitive to pFUS.

This study has several limitations. First, *in vivo* studies will be necessary to confirm that mechanotransductive effects of pFUS observed in suspended or glass-plated cells are similar in solid tumors and also to clarify the explicit roles of both H_2_O_2_ formation and DNA damage in the shift toward proinflammatory TME *in vivo*. Our previous studies 19, 20 did not examine H_2_O_2_ formation or neutrophil infiltration, but the role of H_2_O_2_ in pro-inflammatory TME shifts after pFUS could be due to neutrophil recruitment in addition to DNA damage. Another limitation is that only four tumor cell lines were evaluated. It is unclear how numerous other cancer cell types would respond to pFUS-induced Ca^2+^ fluxes regarding oxidative stress and DNA damage. Investigating additional tumor lines may provide further understanding of the mechanobiology induced by pFUS and its therapeutic potential. Lastly, pFUS in this study did not initiate apoptosis in the tumor cells. Future studies will need to examine the effects of pFUS on cell cycle progression (*e.g.* inducing senescence) and proliferation which could also reduce tumor growth rates *in vivo*.

In conclusion, *in vitro* cell culture models have demonstrated that pFUS generates downstream biological effects through cytosolic Ca^2+^ influx that resulted in mitochondrial ROS formation and double-strand DNA damage in the absence of apoptosis. This study investigated several tumor types and observed heterogenous pFUS responses between cell lines. Furthermore, it suggests differences in pFUS effects arise from specific physiological differences and could provide direction for optimal implementation and translation of pFUS into clinical trials.

## Supplementary Material

Supplementary figures.

## Figures and Tables

**Figure 6:**
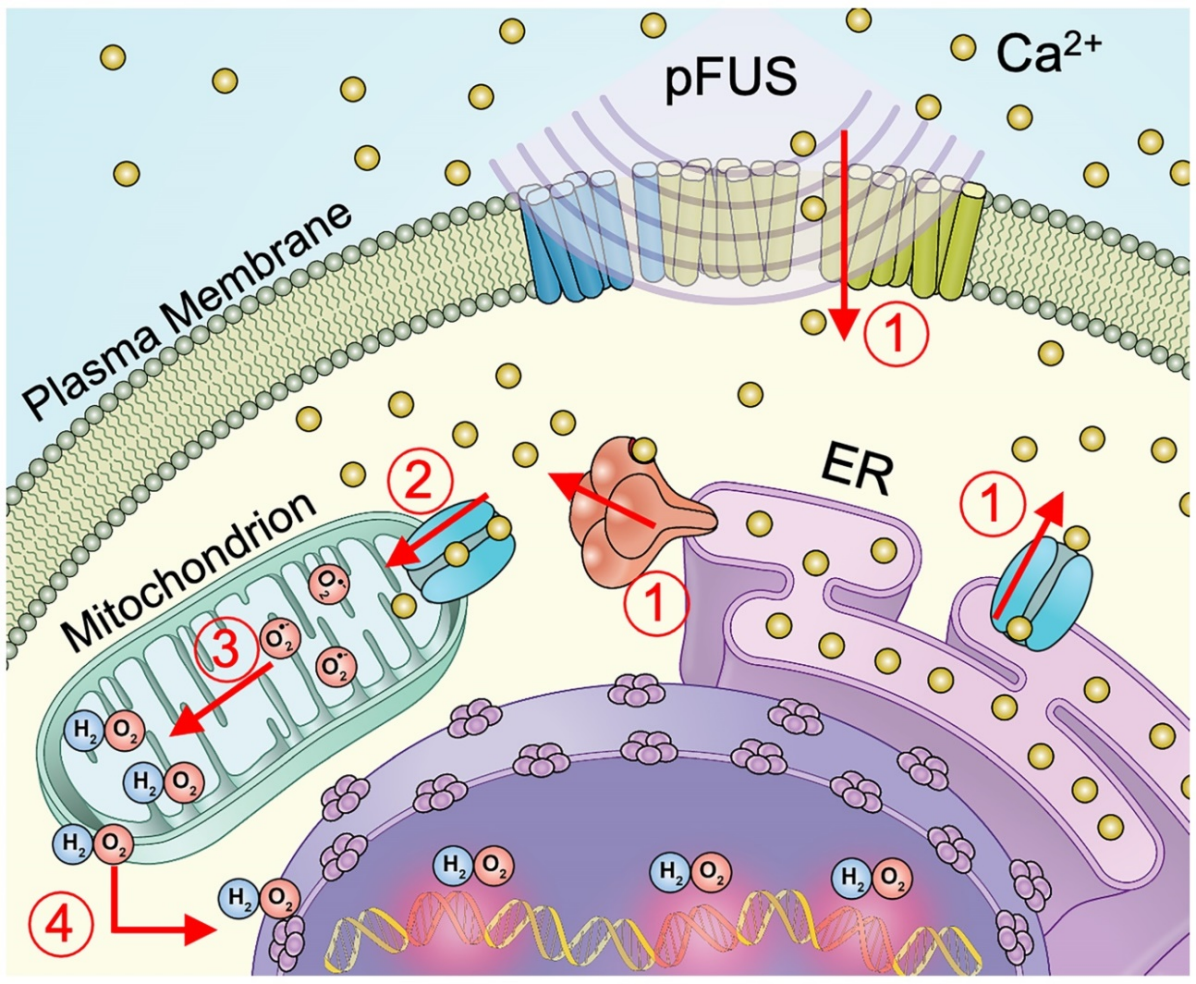
** Schematic representation of cellular Ca^2+^ and ROS dynamics from pFUS to tumor cells.** 1) pFUS causes increases in cytosolic Ca^2+^. Both plasma-membrane and store-release mechanisms (i.e., CICR and IP_3_-mediated release) are hypothesized to be involved. 2) Elevated cytosolic Ca^2+^ levels increase mitochondrial Ca^2+^ influx through the MCU leading to increased superoxide formation. 3) Reduction of superoxide forms H_2_O_2_ which 4) diffuses from the mitochondria to induce nuclear DNA damage.
