# Supplementary material for: Cytosolic Ca2+ transients during pulsed focused ultrasound generate reactive oxygen species and cause DNA damage in tumor cells
Source: Theranostics. 2021 Jan 1;11(2):602–13. doi: 10.7150/thno.48353 (PMC7738866; doi:10.7150/thno.48353)
Supplement: Supplementary file 1 — Supplementary figures. [file thnov11p0602s1.pdf]

Supplemental Information for:

Cytosolic Ca^{2+} transients during pulsed focused ultrasound generate reactive oxygen species and cause DNA damage in tumor cells

Robert B. Rosenblatt[1], Joseph A. Frank[1, 2], and Scott R. Burks[1,*]

[1]Frank Laboratory, Department of Radiology and Imaging Sciences, NIH Clinical Center, Bethesda, MD, 20892

[2]National Institute of Biomedical Imaging and Bioengineering, Bethesda, MD 20892

*Address Correspondence to:

Scott R. Burks, Ph.D.

10 Center Dr., RmB1N256

Bethesda, MD 20892

Ph: (301) 594-2368

scott.burks@nih.gov

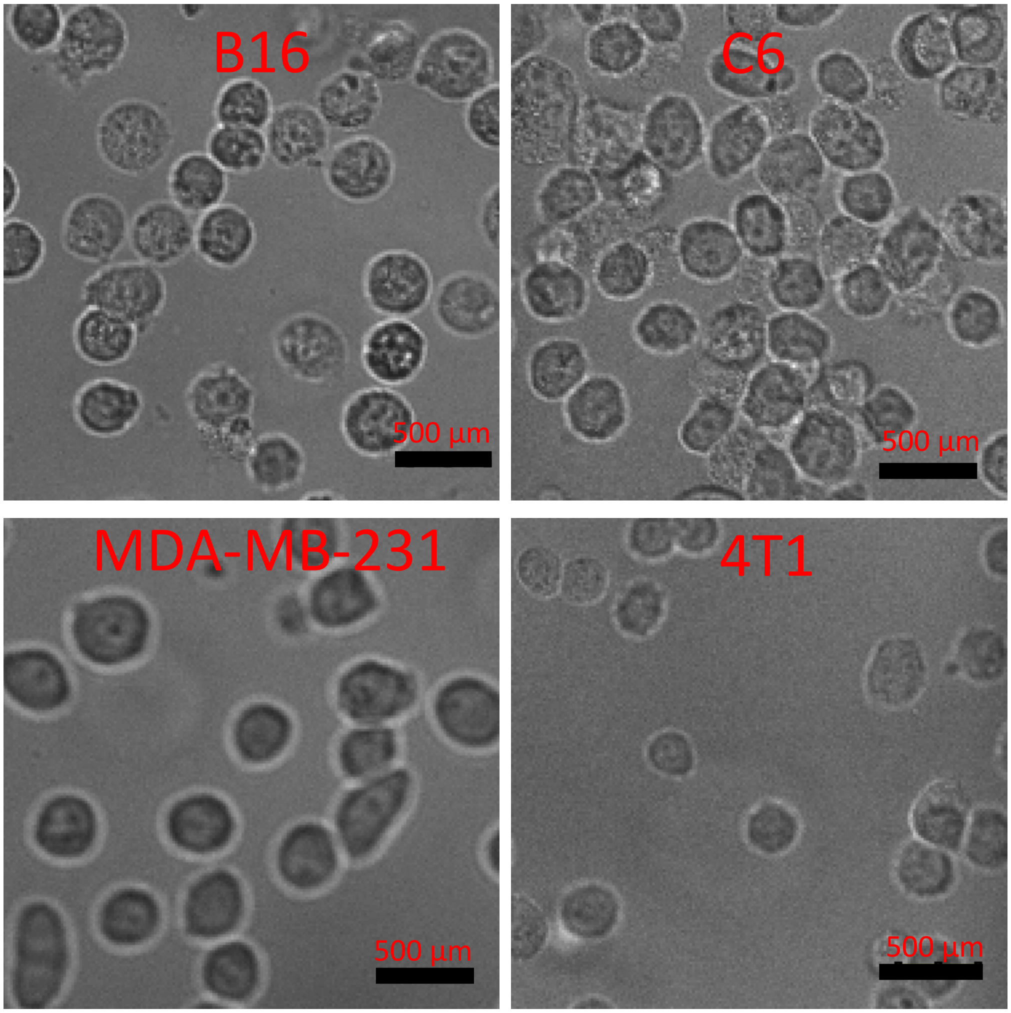

Cell Line	Average Cell Diameter (µM)	SD
B16	20.97	1.18
C6	16.87	2.35
MDA-MB-231	27.17	3.98
4T1	26.07	1.86

Supplemental Figure 1. Means and variance of cell diameters to estimate intracellular volumes. Diameters (n = 100 per cell type) were measured across 3 plates of cells for each type and then used to calculate spherical volumes of cells using the equation: $V = \frac{4}{3}\pi r^2$ where V is volume in μm^3 and r is radius in µm.

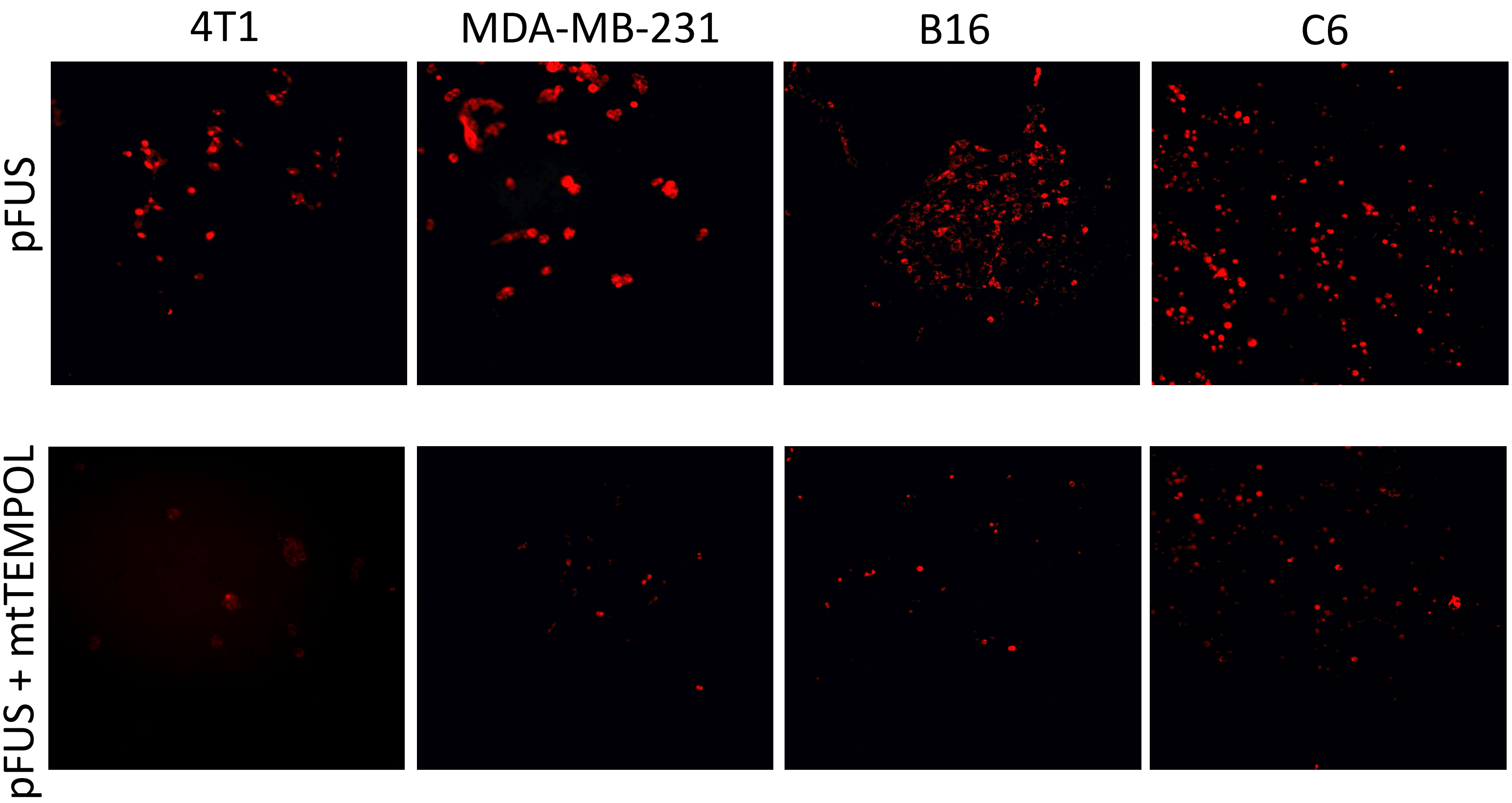

Supplemental Figure 2. mtTEMPOL neutralizes superoxide formation following pFUS to tumor cells. Cells were given pFUS in the presence or absence of mtTEMPOL (20 μM) and allowed to incubate for 2 hr before live cell imaging on an epifluorescent microscope. Measurements were repeated in triplicate with similar results.
